# Risk factors associated with knife-crime in United Kingdom among young people aged 10–24 years: a systematic review

**DOI:** 10.1186/s12889-020-09498-4

**Published:** 2020-09-25

**Authors:** Sara Haylock, Talia Boshari, Emma C. Alexander, Ameeta Kumar, Logan Manikam, Richard Pinder

**Affiliations:** 1 Department of Primary Care and Public Health, School of Public Health, Imperial College London, London, UK; 2 Southwark Council, 160 Tooley Street, London, UK; 3 London North West University Healthcare NHS Trust, London, UK; 4 Aceso Global Health Consultants Limited, London, UK; 5 Faculty of Medicine, University of Southampton, Southampton, UK; 6 UCL Institute of Epidemiology and Health Care, University College London, London, UK

**Keywords:** Weapon-related crime, Youth violence, Knife crime, Risk factors, Gang violence, Gang membership, Adolescent behaviour

## Abstract

**Background:**

Since 2013, the number of violent crimes and offences by sharp instruments have increased continually, following a previous decrease, with majority of cases occurring among young people and in London. There is limited understanding surrounding the drivers influencing this change in trends, with mostly American-based research identifying risk factors.

**Methods:**

The aim of this review is to identify and synthesise evidence from a range of literature to identify risk factors associated with weapon-related crime, for young people (aged 10–24 years) within the UK.

A search strategy was generated to conduct a systematic search of published and grey literature within four databases (EMBASE, Medline, PsycINFO, and OpenGrey), identifying papers within a UK-context. Abstracts and full texts were screened by two independent reviewers to assess eligibility for inclusion, namely study focus in line with the objectives of the review. Weight of Evidence approach was utilised to assess paper quality, resulting in inclusion of 16 papers. Thematic analysis was conducted for studies to identity and categorise risk factors according to the WHO ecological model.

**Results:**

No association was found between gender or ethnicity and youth violence, contrasting current understanding shown within media. Multiple research papers identified adverse childhood experiences and poor mental health as positively associated with youth and gang violence. It was suggested that community and societal risk factors, such as discrimination and economic inequality, were frequently linked to youth violence.

A small number of studies were included within the review as this is a growing field of research, which may have led to a constrained number of risk factors identified. Due to heterogeneity of studies, a meta-analysis could not be conducted. As many studies displayed positive results, publication bias may be present.

**Conclusions:**

Several risk factors were identified, with evidence currently heterogeneous with minimal high-quality studies. However, findings highlight key areas for future research, including the link between poor mental health and knife-crime, and the trajectory into gangs. Risk factors should help identify high-risk individuals, targeting them within mitigation strategies to prevent involvement within crime. This should contribute to efforts aimed at reducing the rising crime rates within UK.

**Systematic review registration number:**

CRD42019138545.

Registered at PROSPSERO: 16/08/2019.

## Background

**Fig. 1:**
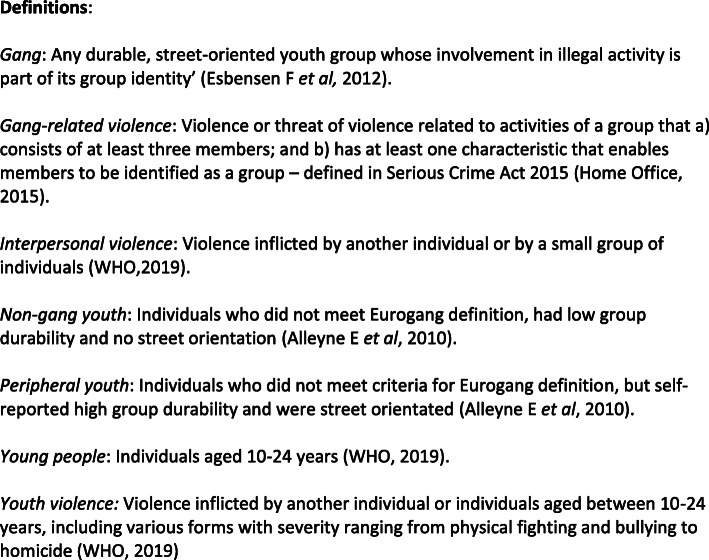
Definitions of key terms mentioned within the review

Youth violence, as defined in Fig. 1, has been increasing globally resulting in substantial economic, social, and psychological costs. Globally interpersonal violence and homicide is the third leading cause of death for 15–19 year olds [1] and although during the 49th World Health Assembly this was declared as a major, worldwide, and increasing issue, strategies aimed to reduce youth violence are yet to be prioritised and implemented.

Youth violence is a particularly pertinent issue in the United Kingdom. According to police-recorded data, the United Kingdom (UK) has seen increasing incidence of youth violence since 2012/13 [2], contrasting with a preceding period of improvement observed globally over the years 2000–12 [3]. A significant trend has been the rise in weapon-related crime, with 285 homicides committed involving a knife or sharp instrument in year ending March 2018 - an increase of 70 offences compared to the previous year [1]. Recent data from the Office for National Statistics revealed an 16% increase in the number of offences involving a knife or sharp instrument in the year ending March 2018 (*n* = 40,147). This figure is suspected to underestimate the actual number of incidents due to issues of record identification from the Greater Manchester Police. Furthermore, in 2017, the most common form of homicide was by sharp instrument with 39,598 offences (a 22% increase since 2016 and 55% increase since 2014) [4].

A large number of young people involved in violence are dying as a result of sharp instruments. Data also shows young people are disproportionately affected by weapon-related crime. The number of homicide victims for the age group 0–24 years is consistently the highest and continues to increase in contrast to all other age groups (excluding 35–44 years) which have remained stable [2]. Similar trends have been observed regarding weapon possession, specifically ‘articles with a blade or point’ [4]. This has resulted in a 55% rise in the number of hospital admissions involving young people in England for assaults involving a sharp instrument since 2012/13 [5], therefore displaying the impact on health services, individuals, and the wider community.

Location also seems to influence this public health issue. For example, London accounts for 48% of the increase in weapon-related crime [2] and recent data also displays a contrast of the number of offences between metropolitan and non-metropolitan areas. This variability may reflect disparities in socioeconomic status, education, availability of weapons, and crime levels. For example, deprivation is shown to contribute to violent crime, as risk of victimisation of those unemployed is double the national average and homicide offenders are most likely to have low socioeconomic status (SES) [6].

Gang violence has received significant attention within the media and is often described as the driving factor behind the rise in knife crime in London [7]. In 2007/08, 55 young people aged 13–19 years died in violent circumstances and according to MPS more than half of these were gang-related [8]. In early 2019, media reports of stabbings and homicides increased dramatically, creating confusion surrounding gangs and their characteristics. For example, in March *BBC News* reported 5 gang members were arrested for knife crime within a school [9]. Also, young males of ethnic minority groups, such as African-Caribbean, along with immigrants and asylum seekers are described as causing the majority of youth violence [10], although associations are yet to be investigated.

Gangs are not wholly responsible for this recent surge in youth violence and the contribution by gangs is difficult to quantify as there is no precise or legal definition of a ‘gang’. Although similarities exist between two commonly used definitions - displayed in figure one – there are clear differences. The link between gang violence and violent crime requires further investigation.

Despite the rising tide of weapon-related crime, minimal research has been conducted within a UK-context with the majority of research, displayed by the WHO, guiding mitigation strategies based on American gangs and associated violence. The social and legal context of these two countries differ, particularly around availability and use of firearms. Therefore, this research has limited use [11]. Multiple risk factors have been identified, including but not limited to: race, gender, gang membership, deprivation, social media, and adverse childhood experiences (ACE). However, risk factors lack clarity, are yet to be collectively analysed, and require a systematic assessment and evaluation.

## Methods

### Research question

What are the risk and protective factors relating to the rise in knife crime associated with weapon carrying, weapon usage, homicides, gang involvement, or victimisation of weapon-related crime, for young people (aged 10–24 years) within the UK?

### Aim

The aim of this systematic review is to identify and synthesise evidence from a wide range of literature to identify risk or protective factors associated with weapon carrying, weapon usage, homicides, gang involvement, or victimisation of weapon-related crime, for young people (aged 10–24 years) within the UK.

### Rationale

There has been a clear and consistent rise in police-recorded incidents involving a knife or sharp instrument in the UK since 2014 [5], with weapon-crime described as a new epidemic [12]. This challenge requires a holistic approach and Public Health would provide a central role in characterising responses and providing leadership. Relying entirely on law enforcement would be inefficient. However, the complexity of weapon-related crime and associated risk factors is not completely understood as minimal research has investigated these risk factors within a UK setting. This lack of research has led to misleading media reporting and no consistent or strategic approach to tackle this growing problem. Through identifying common risk factors, interventions can be aimed at those vulnerable to future involvement within violent crime, acting as a preventative method. Differences between gang members and non-gang youths also need to be highlighted as this may help inform timely interventions, reaching youths before they engage in gang activities.

This review focuses exclusively on young people aged 10–24 years given the significant increase in victimisation and involvement within violent crimes compared to any other age group, introduced previously [4, 12].

This systematic review is the first paper to combine information, from published literature, of youth violence and associated risk factors within a UK context. This paper aims to provide essential evidence, directing future interventions to effectively reduce weapon-related crime and understanding the trajectory of individuals into violent crime and - in some cases - gang membership.

### Protocol

The protocol is registered to PROSPERO (CRD42019138545, https://www.crd.york.ac.uk/PROSPERO/) and is reported according to PRISMA guidelines.

### Search strategy

**Fig. 2:**
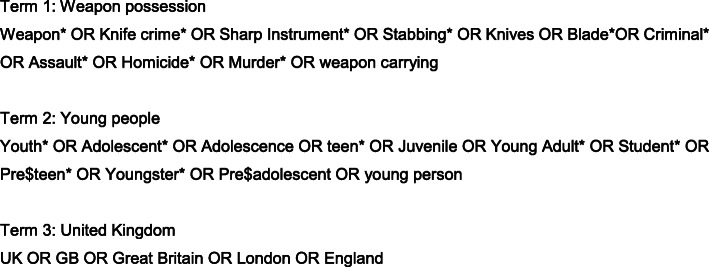
Search strategy used to identify papers within academic databases. Full search strategies can be available from authors on request.

A research phase enabled the development of search terms (Fig. 2), specific to study setting, which were entered into EMBASE, Medline, and PsycINFO. Keywords from each term were combined to further narrow results, ensuring relevant articles were identified, for example searching ‘adolescent’ AND ‘knife crime’ AND ‘United Kingdom’. Using terms such as ‘youth violence’ and ‘knife crime’, a search was performed on the database OpenGrey to include grey literature, thus investigating information beyond published articles. There was limited capability to form a structured search on OpenGrey, therefore a search string similar to the search strategy used for research databases was not possible. English was set as a language limit, removing articles without translation available. An example search strategy can be found in Appendix A.

### Inclusion criteria

Papers were included within the review if they met the following criteria, ensuring risk factors were specifically associated with weapon-related crime within the UK:

A Paper identified risk or protective factors associated with weapon carrying, weapon usage, homicides, gang involvement, or victimisation of weapon-related crime;
B Study participants included young people (aged 10–24 years);
C Article setting was within United Kingdom;
D The paper was a form of published paper, grey literature, conference abstract, or unpublished thesis.

Articles were excluded if:

A Population did not look specifically at young people (aged 10–24 years);
B Papers identified risk factors associated with sexual violence or other violent/victim-based crime (as the review focuses on risk factors associated with crime involving knives or sharp instruments and not associated with any other crime);
C Papers identified special education needs/drug or alcohol misuse as risk factors as these associations have already been well-reported throughout previous research;
D English translation was not available;
E Papers were published prior 1990 in order to ensure relevance.

## Results

### Study selection, data extraction and analysis

A two-stage screening process was conducted, identifying articles eligible for study involvement. Initially, titles and abstracts were reviewed independently by two reviewers and, if meeting inclusion criteria, articles were deemed eligible for the second phase of screening. Full texts were assessed against criteria to evaluate whether papers were suitable for study inclusion. Any discrepancies in screening or data extraction were discussed until a consensus was reached.

As part of a narrative synthesis a deductive thematic analysis [13] was conducted on studies to extract and categorise risk factors, in-line with the WHO Ecological Framework, used to guide violence prevention strategies [14]. The model was generated to display the complexity of risk factors associated with interpersonal violence, and how this outcome is an interaction of factors across a variety of levels [14]. The ecological model classifies risk factors into four main groups: individual, relationships, community, and societal – each category is further broken down by the WHO, to provide a list of key variables, and are displayed in Table 2 [14]. The categories within this model were used when interpreting the review’s findings. It was expected that the majority of risk factors identified within studies would align with the WHO framework. However, certain risk factors mentioned in the model were not captured within included studies (displayed in Table 2). This may be a result of the limited number of studies included within the review.

During the preliminary synthesis, the thematic analysis was conducted as papers were analysed by searching for risk factors that aligned with the themes developed from the existing WHO concepts [14] (Table 2). This was used to identify any patterns, similarities or differences of risk factors across the included studies. When identified, the primary author coded risk factors by colour and inputted into a spreadsheet. These were then tabulated to organise, present, and count findings following the WHO framework. Paragraphs were then drafted based on the frequency that a risk factor was mentioned across the studies. For example, seven studies highlighted ‘adverse childhood experiences’ as a risk factor, therefore these results were further explained. By using pre-determined themes, this research aims to guide recommendations based on a variety of studies, ensuring results can be utilised for future public health policies.

Meta-analysis was not conducted due to study heterogeneity in methodology and focus. Heterogeneity was assessed in the included studies for: clinical heterogeneity (the varied participant groups and outcomes assessed); and methodological heterogeneity (the differing WOE scores (Table 1) as well as study design, and statistical tests performed). Diversity of study is portrayed in Appendix B. This contains a summary table presenting key characteristics of included studies, providing information about study design, population, outcomes, and statistical tests when appropriate. This was reported prior to the preliminary synthesis.

**Table 1:** Results of the quality analysis of papers. Table displays results from quality analysis, using WOE approach, of papers meeting the systematic review criteria.

Author, Year, and Title	A	B	C	D (Overall)
Densley, JA et al. 2015 We’ll show you gang’: The subterranean structuration of gang life in London	M	M	H	M
Hansen, K. 2003 Education and the Crime-Age Profile	M	L	M	L
Alleyne, E et al. 2014 Denying humanness to victims: How gang members justify violent behavior.	H	M	M	M
Smith, D. 2007 An investigation into causal links between victimization and offending in adolescents.	H	M	M	M
Nasr, IN et al. 2010 Gender inequality in the risk of violence: material deprivation is linked to higher risk for adolescent girls.	H	M	L	M
Hayden, C. 2010 Offending behaviour in care: is children’s residential care a ‘criminogenic’ environment?	H	M	M	M
Falshaw, L et al. 1997 Adverse childhood experiences and violent acts of young people in secure accommodation	H	M	M	M
Alleyne, E et al. 2016 Psychological and behavioural characteristics that distinguish street gang members in custody	H	M	M	M
Bailey, S et al. 2006 The social background and nature of “children” who perpetrate violent crimes: A UK perspective.	H	M	L	M
Wood, JL et al. 2017 Differentiating Gang Members, Gang Affiliates, and Violent Men on Their Psychiatric Morbidity and Traumatic Experiences.	H	H	M	H
Barlas, J et al. 2006 Weapons carrying in British teenagers: The role of personality, delinquency, sensational interests, and mating effort.	H	H	H	H
Briggs, D. 2010 ‘True stories from bare times on road’: Developing empowerment, identity and social capital among urban minority ethnic young people in London, UK.	H	M	M	M
Densley, J et al. 2011 Ganging up on gangs: Why the gang intervention industry needs an intervention.	H	H	H	H
Alleyne, E et al. 2010 Gang involvement: psychological and behavioral characteristics of gang members, peripheral youth, and nongang youth.	H	H	H	H

To assess the robustness of the narrative synthesis, the primary research articles included were quality appraised, at study level, by the primary author using the Weight of Evidence approach (WOE) [15]. This method was selected as a variety of different study designs were collected with a range of information available. This process allows for integration of evidence obtained from various results and methodologies when answering the proposed research question. This form of analysis assesses the overall quality of each paper depending on the following three criteria: WOE A (used to examine the clarity and accuracy of information); WOE B (assessing appropriateness of study methodology); and WOE C (how relevant study findings are to this systematic review). For each study, these three judgements were then combined to provide WOE D: an overall assessment of quality and relevance of evidence for risk factors associated with weapon usage and knife crime (Table 1). Research papers were ranked as either high (H), medium (M), or low (L) and were included only if overall quality (WOE D) rated as high or medium. These rankings were used to evaluate strength of key findings and results. Where there was uncertainty concerning study quality following the above criteria, the primary author liaised with co-authors to clarify ranking. This aimed to ensure reliability of the quality appraisal.

The Synthesis Without Meta-analysis (SWiM) guideline [16] was used to ensure clarity when reporting methods, including the narrative synthesis, and results of the review. A supplementary table outlining the checklist and where to find the reported information within the manuscript can be found in Appendix C.

**Fig. 3:**
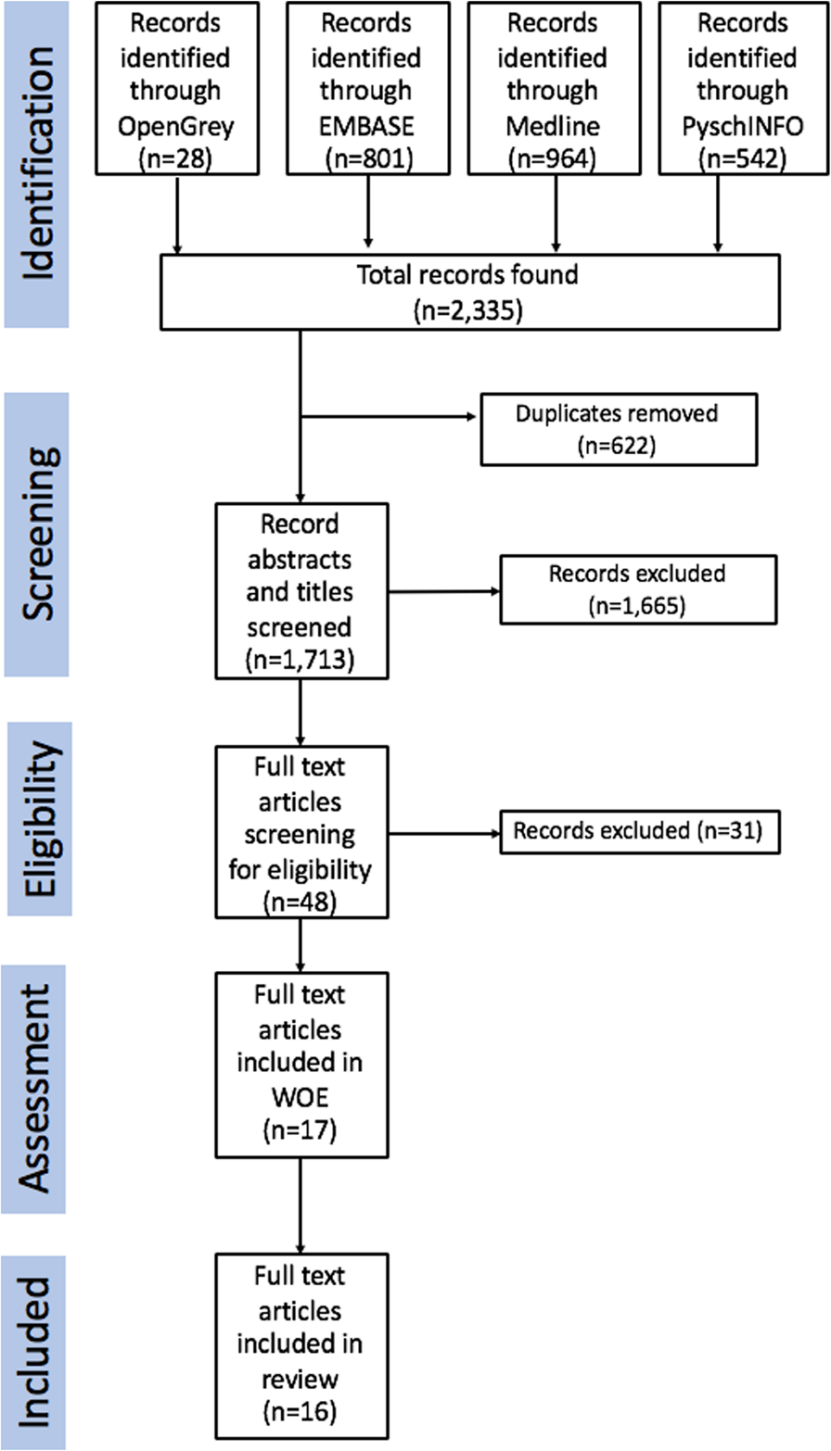
PRISMA flowchart displaying the number of articles found from each database (2,335), number of duplicates removed (622), papers removed from abstract screening (1,665), and removed from full text screening (31).

A total of 2335 articles were originally identified from the initial search (PRISMA flowchart (Fig. 3)), from which 622 duplicates were removed, resulting in 1713 articles taken forward for abstract review. Stage one review excluded a further 1665 articles and stage two, 31. Seventeen articles were included in the WOE quality assessment, where only one paper was excluded from the review with a quality ranked as low (L). Therefore, 16 articles were included in the systematic review. A quantitative analysis was not undertaken due to the heterogeneity of studies included (Appendix B), and therefore a narrative synthesis of risk factors influencing weapon-related crime was conducted.

### Study characteristics

Of the 16 articles included, one was an intervention study evaluating current mitigation strategies for gang-related crime in London [8], while all other studies were observational. Included within the review were: three literature reviews [17–19]; three cohort studies [20–22]; three qualitative interview studies [8, 23, 24]; three case-control studies [25–27] 2017; and four cross-sectional studies [28–31]. All studies were conducted within the UK: four within the UK; one in Birmingham; one in Edinburgh; five in London; one in Wales; and one in England. Appendix B presents detail characteristics of each study, including study size and key findings.

To provide a rich reflection of evidence for the reader, we included both quantitative and qualitative studies as there is comparatively little evidence published on this topic within the inclusion parameters specified. Deductive thematic analysis was completed for qualitative papers using themes as set-out by the WHO. Therefore, if these themes/risk factors were identified within the paper, the paper was incorporated into Table 2 irrespective of study design.

**Table 2:** Summary of risk factors included studies. Displays which risk factors - associated with youth violence - are described in papers and whether there is a positive, no, or unclear association. For papers including a quantitative analysis, risk factors with statistically significant results were categorised as positively associated. Whereas for qualitative studies, risk factors mentioned within interviews or literature reviews were identified as positively associated. The design of each study is further highlighted within the table. The count of studies mentioning each risk factor was used to determine the relevance for discussion

Individual risk factors	Positive association	No association	Unclear association
**Aged between 10 and 24 years**	**6 studies** **Quantitative** Alleyne et al*,* 2010; Alleyne et al*,* 2014; Barlas et al, 2006; Falshaw et al, 1997; Hayden et al, 2010; **Qualitative** Densley et al*,* 2015;	**0 studies**	**0 studies**
**Gender**	**3 studies** **Quantitative** **Males** Falshaw et al, 1997; Nasr et al, 2010; **Females** Hayden et al, 2010;	**3 studies** **Quantitative** Alleyne et al*,* 2010; Alleyne et al*,* 2014; **Qualitative** Briggs et al*,* 2009	**1 study** **Quantitative** Barlas et al*,* 2006;
**Ethnicity**	**1 study** **Quantitative** Smith I et al*,* 2007;	**3 studies** **Quantitative** Alleyne et al*,* 2010; Alleyne et al*,* 2016; Smith D et al*,* 2007;	**0 studies**
**Education**	**3 studies** **Quantitative** Bailey et al*,* 2001; Clement et al, 2010; Hayden et al, 2010;	**0 studies**	**1 study** **Qualitative** Briggs et al, 2010
**Adverse childhood experiences**	**7 studies** **Quantitative** Alleyne et al*,* 2010; Bailey et al, 2001; Falshaw et al, 1997; Smith D et al*,* 2007; Smith I et al*,* 2007; Wood J et al*,* 2017; **Qualitative** Briggs et al, 2010;	**0 studies**	**0 studies**
**Poor mental health**	**3 studies** **Quantitative** Bailey et al, 2001; Barlas et al*,* 2006; Wood J et al*,* 2017;	**0 studies**	**0 studies**
**Previous victimisation**	**3 studies** **Quantitative** Barlas et al*,* 2006; Smith D et al*,* 2007; Wood J et al*,* 2017;	**0 studies**	**0 studies**
**History of violent behaviour**	Not identified	Not identified	Not identified
**Relationship risk factors**	**Positive association**	**No association**	**Unclear association**
**Poor parental attachment**	**2 studies** **Quantitative** Nasr et al, 2010; Smith D et al*,* 2007;	**0 studies**	**0 studies**
**High-risk peer groups**	**7 studies** **Quantitative** Alleyne et al*,* 2010; Alleyne et al*,* 2016; Barlas et al*,* 2006; Falshaw et al, 1997; Hayden et al, 2010; Smith D et al*,* 2007; **Qualitative** Briggs et al, 2010;	**0 studies**	**0 studies**
**Marital Discord**	Not identified	Not identified	Not identified
**Low socioeconomic household status**	Not identified	Not identified	Not identified
**Community risk factors**	**Positive association**	**No association**	**Unclear association**
**Deprivation/high rates of unemployment**	**6 studies** **Quantitative** Alleyne et al*,* 2010; Alleyne et al*,* 2016; Nasr et al, 2010: Wood R, et al 2010; **Qualitative** Briggs et al, 2010; Densley et al*,* 2015;	**0 studies**	**0 studies**
**High crime levels**	**1 study** **Quantitative** Wood R, et al 2010;	**0 studies**	**0 studies**
**Low social cohesion**	**1 study** **Quantitative** Barlas et al*,* 2006;	**0 studies**	**0 studies**
**High residential mobility**	Not identified	Not identified	Not identified
**Societal risk factors**	**Positive association**	**No association**	**Unclear association**
**Economic inequality**	**3 studies** **Quantitative** Nasr et al, 2010; Wood R, et al 2010; **Qualitative** Densley et al*,* 2015;	**0 studies**	**0 studies**
**Marginalisation/** **stigma/** **discrimination**	**4 studies** **Quantitative** Alleyne et al*,* 2010; Alleyne et al*,* 2016; **Qualitative** Densley et al*,* 2011; Densley et al*,* 2015;	**0 studies**	**0 studies**
**Perception of status and masculinity**	**6 studies** **Quantitative** Alleyne et al*,* 2010; Alleyne et al*,* 2014; Alleyne et al*,* 2016; Barlas et al*,* 2006; Clement et al, 2010; **Qualitative** Briggs et al, 2010;	**0 studies**	**0 studies**
**Relationship with police**	**3 studies** **Quantitative** Alleyne et al*,* 2010; Alleyne et al*,* 2016; **Qualitative** Densley et al, 2010;	**0 studies**	**0 studies**
**Rapid Social Change**	Not identified	Not identified	Not identified
**Cultural norms**	Not identified	Not identified	Not identified
**Gender Inequalities**	Not identified	Not identified	Not identified

As previously mentioned, risk factors were divided into four categories, and subcategories within these, following the Ecological Framework put forward by the WHO [3]: individual; relationships; community; and societal. WHO violence risk factors not identified within included studies are also shown. Each subcategory of risk factor is discussed in the subsequent sections. As mentioned previously, a paper focusing on education and crime age profile was not included within the review due to ranking low quality [32].

### Individual risk factors

#### Demography

Of the 16 studies included within the review, 10 investigated the association of demography with knife crime (Table 2). All six studies investigating age found a positive association between knife crime and adolescence [21, 23, 26, 28, 30, 31]. A 2006 cross-sectional study, using the Juvenile Attitudes Towards Weapon Scale (derived from the Attitudes Toward Guns and Violence Questionnaire), found that the prevalence of weapon carrying increases with age: 30% for individuals aged 11–13 years, 38.2% at 14–15, 47.4% at 16–17, and 52.6% at ages 18–19 [30] (WOE = H). Regarding gang violence, three studies found a young person, compared to other age groups, is positively associated with being in a gang [23, 26, 33]. Densley et al (WOE = M) interviewed 69 self-described gang members, recruited from six London boroughs experiencing high levels of socioeconomic deprivation, with an age range of 13–34 (mean age 20). A participant aged 25 years described gang life to be a ‘young man’s game’ and ‘when you’re younger it’s about what you’ve got now and how fast…when you’re older you’ve got more to lose’ [23].

Results were mixed regarding the association with gender. One cohort and one cross-sectional study showed males were more likely to be associated with knife crime [22, 28]. However, a cohort study of in care homes suggested females were more likely to offend at a younger age [21](WOE = M). A cross sectional-study looked more closely at the characteristics of weapon-carrying, e.g. type and use of weapon, and found no significant difference on the basis of gender: although 27% of males used their weapon to injure compared to 19% of females [30]. No significant association between gang violence and gender was found in the three papers exploring this issue [24, 26, 31]. For example, both males and females expressing the need to be the ‘biggest, baddest and the most untouchable’ [26] (WOE = M).

A cohort study found no association between knife crime and the ethnicity of the victim or perpetrator when controlling for confounders, including sex and family structure at an individual level and neighbourhood deprivation at a community level [17](WOE = M). However, a literature review suggests that migrants and refugees may be at higher risk of victimisation of weapon-related crime [20] which may explain the overrepresentation within the media. A cross-sectional study of 797 school students and a case-control with 188 young offenders completed self-reported questionnaires and, through use of pre-determined criteria, were divided into categories depending on gang involvement. Comparisons of a variety of groups, rather than investigating one specific cohort of gang members, allows for differences to be evaluated. As neither studies identified a difference between the ethnicity of groups divided by level of gang involvement, this suggests no association with gang violence [20, 25, 31].

#### Adverse childhood experiences (ACE)

All seven studies investigating the association between teenagers with ACEs and weapon-related crime reported a positive association (Table 2). A cross-sectional study of 20 males convicted of homicide during their adolescence investigating adolescent homicide found that 25% of perpetrators had experienced either sexual or physical abuse and 90% were known to social services [29] (WOE = M), and all 20 had previously experienced neglect or parental separation [29]^;^ a higher prevalence compared to the general population. Within a study based in a care home, 91% of young people who had been convicted of a crime (the majority of which were violent or weapon-enabled) had experienced multiple placements (range of 1–30, mean of 8) [28] (WOE = M). Wood J et al (WOE = H) showed in a cross-sectional study that gang members and ‘gang affiliates’ self-reported more childhood traumatic events and were more likely to have been placed in local authority care compared to violent men not in a gang [27].

#### Education

Three studies investigated the impact of school exclusion on involvement in knife crime [19, 21, 29]. However, one study did not show a clear association between education and gang membership. During qualitative interviews current and previous gang members expressed their opinions that school achievements and successful routes through education were unattainable [24]. On the other hand, some gang members had obtained GCSEs and were still involved in criminality [24].

Clement et al identified in a Bristol-based study that 80% of younger offenders had previously been excluded from school, suggesting a link between school exclusion and involvement in violence [19]. In contrast, Hayden et al found similar rates of school exclusion between offenders and non-offenders within a care home setting (40% for non-offenders and 44% for offenders) [21]. However, as all individuals were removed from their family home this may affect the findings.

#### Mental health

Three studies investigated mental health associated with knife crime and both described poor mental health (suicide/depression/self-harm described by participants) as a risk factor [27, 29, 30]. A cross-sectional study of 20 adolescents committing homicide revealed that all participants suffered from high levels of interpersonal conflict and psychological vulnerabilities [29]. A case-control study of 1539 men found that self-identified gang members and gang affiliates had a higher prevalence of psychological issues, including anxiety, psychosis and suicide attempt, than violent men not involved in gangs [27]. For the particular study, gang affiliates and members both were involved in gang-related activity, however, categories differed as gang affiliates did not identify as a gang member.

#### Victimisation

Three studies investigated a link between previous victimisation of weapon-related crime and offending [20, 27, 30] that among young people self-reporting weapon possession within the last 6 months, ‘reactive weapon carriers’ - where weapon was used in conflict resolution, or user was a victim of threat, and/or injury - reported previously being a victim of threat or injury. Smith D et al (WOE = M) found that victims of bulling were also more likely to offend and be victims of weapon-related crime. Furthermore, gang members were more likely to be targeted as victims and self-reported more serious injuries compared to non-gang members [30]. Gang affiliates also reported more incidents involving physical attacks compared to violent men who were not part of gangs, however unexpectedly more than gang members [27]. Results found individuals who self-reported victimisation were more likely to offend and vice versa, therefore a bidirectional relationship may exist between being a perpetrator and victim of weapon-related crime.

### Relationships risk factors

Seven studies reported peer influence as an important risk factor for knife crime (Table 2). For example, Barlas et al (WOE = H) described self-identified ‘offensive weapon carriers’ – those who carry weapons to injure or threaten – considered peer influence an important reason for weapon carrying. In conjunction with this, two studies showed peer influence as an important risk factor for gang membership [25, 26]. A majority (65%) of self-described gang members, and 57% of self-described members who also met Eurogang definition, identified one reason for joining was ‘because a friend was a member of the group’ [25].

Two studies investigated parental relationships in association with knife crime and all identified that strong parental attachment acted as a protective factor [20, 22]. At age 15, a cohort study found conflicts with parents increased risk of victimisation and offending [20].

#### Community

Six studies included in this review investigated the impact of deprivation, all of which showed a positive association with knife crime. During interviews, Densley et al (WOE = M) concluded that areas with low socioeconomic status increase risk of gang involvement and with one interviewed gang member describing London communities as ‘built to encourage crime’ [23]. According to a literature review, crime rates are highest in areas of economic deprivation, increasing the chance of adolescents’ involvement within violent crimes [18]. Alongside this, individuals who identified as ‘defensive weapon carriers’ expressed the need to carry weapons for personal safety in high crime areas [30]. Deprivation can further result in low social cohesion which has further been associated with offending behaviour of adolescents and gang members [31].

#### Societal

Three studies suggested a positive impact of economic deprivation and knife crime (Table 3). Densely et al explains how economic inequalities have forced young men into ‘self-destructive behaviour’ as the societal problems have left individuals with minimal options [23]. Four studies showed a positive association between stigma and discrimination and weapon-related crime [8, 23, 25, 31]. A cross-sectional study of 797 secondary school students found negative perceptions of authority were highest in gang members, followed by peripheral youth (individuals involved within gang-related activity, but not classified as members), and lowest in non-gang youth [31]. Furthermore, within interviews gang members described themselves as ‘urban outcasts’, explaining that ‘[they’re] automatically stereotyped, it’s like all black people are criminals… after a time you feel like ‘oh we a gang now? Ok we’ll show you gang’ [23].

Three studies described violence and weapon carrying as a method of gaining status, power, and masculinity [19, 30, 31]. For example, Barlas et al (WOE = H) found young people explained the most common reasons for weapon carrying were: ‘for looking cool’, ‘other people’s respect’, ‘feeling powerful’, and ‘peer admiration’ [30]. Within five studies, gangs were described as providing identity, status, and companionship with membership proving as a method to build an individual’s reputation [19, 24–26, 31]. For example, gang members have expressed the desire to ‘win approval from peers’ and two studies found that young gang members perceived social status as more important compared to non-gang-involved adolescents [19, 30].

## Discussion

Violence is a complex issue as many risk factors are interlinked, thus determining each predictor’s overall influence difficult to characterise. However, the results of the systematic review suggest an unstable environment - within a family, community, or society setting – derived from a multitude of risk factors is a key driver for involvement in weapon-related crime. This is the first systematic review to assess a wide range of literature to identify risk factors for weapon-related crime, collating and analysing information surrounding a topical and growing public health issue.

### Ethnicity and community factors

Results did not identify a strong relationship between ethnicity and youth violence when controlling for confounders, such as SES [20, 25, 31], which contrasts information displayed in the media. While ethnicity had no association, community and societal factors such as economic deprivation did, and these characteristics tended to correlate with certain ethnic minorities. For example, results showed migrants and refugees recently entering the UK were at higher risk of victimisation [17] – this may be a result of discrimination these individuals face when entering a new community. It has also been shown that gangs are homogenous and often mirror the demography of the community they associate with [31]. This relationship between risk factors may lead to the overrepresentation of ethnic minorities as perpetrators and victims of weapon-related crime within police-recorded data and the media.

### Gender

This systematic review did not reveal a clear association between gender and youth violence. The societal pressures of males to display masculinity may provide a possible explanation for their increased threatening behaviour [33]. Research papers investigating the link between gender and weapon-related crime have shown there are multiple aspects of behaviour regarding knife crime, for example ownership, type of weapon, and use. However, due to the mixed evidence in this review alone, it is not possible to confirm this relationship or if gender is a risk factor.

### Adverse childhood experiences

Seven studies identified ACEs as significant risk factors for weapon-related crime, which strongly supports the relationship between early childhood trauma and violence. It can be argued that trauma and an unstable family life create an environment which is likely to manifest aggression and poor mental health, increasing the risk of violent behaviour [34]. This coincides with previous knowledge regarding the long-term effects of traumatic childhood on health within adulthood, including economic deprivation, anxiety, and aggression [35–37]. Gangs may also provide a sense of security that is lacking from their family environment and, as mentioned by Public Health England, a sense of belonging which is fundamental for an individual’s social identity [38]. Furthermore, two studies highlighted the protective nature of strong parental attachment [20, 22] which further supports the importance of a stable home environment and may counteract the effects caused by ACEs. Similar results were found in US-based studies with parental monitoring being negatively associated with gang membership, reducing the effect of other risk factors on adolescents [39].

This is further supported by the association between poor mental health and weapon-related crime, identified by Bailey et al, Barlas et al, and Clement et al. Poor mental health may be on the causal pathway from ACEs to violent behaviour as those suffering from trauma are more likely to experience poor mental health [36]. Therefore, these individuals are more likely to act aggressively and those with suicidal thoughts might not consider the repercussions of their actions.

### Strengths and limitations

There is limited understanding of risk factors for weapon-related crime among young people and current knowledge of gangs has mostly been derived from research conducted within the USA, which means findings will be influenced by its environment of high gun ownership and incarceration rates. Within a growing field of research, this paper is the first to collect information from scientific and grey literature, analysing and comparing risk factors for weapon-related crime. Therefore, this review provides essential evidence on risk factors identifying which individuals are at high-risk, directing public health interventions to target those most vulnerable to effectively reduce youth violence. Results are also specific to the UK, with other reports focusing on wider regions, such as Europe, allowing for precise suggestions for mitigation.

However, findings should be balanced against a number of limitations. Only 16 studies were eligible for inclusion, which may have resulted in a constrained range of risk factors identified. However, this is a growing field of research, resulting in a limited number of sources available. Literature reviews were also included within the review and these may be affected by the authors experience or personal views, therefore these should be contextualised. A meta-analysis could not be conducted due to the heterogeneity and types of studies included within the review, therefore a statistical estimate of effect for each risk factor could not be produced. A narrative synthesis was conducted, which may have resulted in unreliability, lack of transparency, and potential reviewer bias as conclusions are based on subjective interpretation [40]. However, due to the substantial heterogeneity in populations, outcome, and methodology, a narrative synthesis was the most appropriate methodology for this review. Although we included grey literature, publication bias is likely to be present, particularly as many studies included within the review conclude positive results. Studies suggesting no association with risk factors and youth violence might by underrepresented within this review.

With regards to the studies included within the review, qualitative interviews investigating gang membership used a chain referral method to recruit participants. This would have inherent bias as only a specific group of individuals are likely to be included within the analysis, potentially only identifying the same risk factors. Self-reported questionnaires were also utilised, which may have resulted in erroneous recall. However, due to the sensitive nature of the topic, these methods may be most appropriate to ensure individuals provide honest and accurate information.

### Comparison with previous literature

The identification of risk factors such as ACEs and poor mental health is in line with previous knowledge as a relationship exists between trauma and involvement within weapon-related crime. For example, multiple studies have highlighted the effect of childhood trauma on adolescent and adult health, psychological and physical [36]. Areas of high crime, violent incidents, low socioeconomic status and the relationship with youth violence have also previously been highlighted within previous worldwide research [3]. However, contrasting previous literature, no significant association was found between gender and youth violence. Reports have suggested females play secondary roles within violent crime and gang activity [10], which may suggest the characteristics of gangs are evolving and research needs updating.

Although many risk factors mentioned within this review have been previously identified, they have not yet been collectively analysed. Therefore, compared to previous literature, this review highlights the interconnected nature of risk factors for weapon-related crime and the necessity for a holistic preventative approach.

### Policy implications

As no clear association was found between gender, ethnicity and weapon-related crime, policy makers should avoid targeting individuals based on stereotypes in these areas. This may also reduce discrimination within policy efforts, ensuring a holistic approach to mitigate youth violence. Individuals with ACEs and mental health issues should be targeted within prevention strategies as results suggest these groups are at high-risk for future involvement within violent crime. Thus far studies investigating this outcome have been very heterogeneous and mixed in quality, further research is necessary in order to aid the design of interventions and to aid policymakers.

To prevent individuals in areas of deprivation using violence as a method to improve social status, it is essential for policy makers to target areas of deprivation when tackling gang crime. Strategies should be aimed at improving employment skills, self-esteem, and also community involvement to increase social cohesion at a young age given the influence of ACEs, acting to prevent future formation of gangs as well as improve the quality of life for the adolescent population.

## Conclusion

Youth violence is an increasing public health issue within the UK and London in particular. This study collected information regarding risk factors from a wide range of sources, uniquely examining them within a UK setting. The review demonstrates the importance of stability for an adolescent during times of vulnerability with each risk factor eroding this sense of security. Although it is important to recognise not all adolescents with these risk factors will commit crimes or engage in gangs or violent behaviour, the identified risk factors can act as warning signs that captures young people before they become victims of violence. This provides essential evidence on which individuals are at high-risk, directing public health interventions to target those most vulnerable to effectively reduce youth violence.

## Data Availability

The papers analysed within the review are available from the corresponding author on reasonable request.

## Appendix 1

Search strategy used for PubMed

Copy and insert the string into the search bar. The asterisk used in the search truncates the search term so that, for instance, both ‘teen’ and ‘teens’ are retrieved.

Boolean operators must be written in capitals (AND, OR).

((((Weapon* OR Knife crime* OR Sharp Instrument* OR Stabbing* OR Knives OR Blade*OR Criminal* OR Assault* OR Homicide* OR Murder* OR weapon carrying)) AND (Youth* OR Adolescent* OR Adolescence OR teen* OR Juvenile OR Young Adult* OR Student* OR Pre$teen* OR Youngster* OR Pre$adolescent OR young person)) AND (UK OR GB OR Great Britain OR London OR England)) AND (UK OR GB OR Great Britain OR London OR England)

## Appendix 2

Table provides list of all studies included, providing information on location, study design, sample size, and participants. A summary of key risk factors identified are also shown. Further quantitative results can be found within the papers referenced

**Table:**

Author, Date, and Title	Location	Study Design	Sample Size and Participants	Statistical Test	Risk Factors and Indicators
Smith, ID 2007 Being tough on the causes of crime: Tackling family breakdown to prevent youth crime	London	Literature review	N/A	N/A	**Parental separation**: 70% of young offenders come from lone-parent families In 2 parent families, children have more access to attention **Dysfunction** (parents not able to provide nurturing environment): Children likely to develop psychological disorders **Fatherlessness**: Paternal role is more than financial resource
Wood, R 2010 UK: the reality behind the ‘knife crime’ debate	UK	Literature review	N/A	N/A	Comparing London to Greater London **Social inequality** increases chance of involvement in youth violence Identifies differences in **ethnicity** of offenders depending on location
Clement, M 2010 Teenagers under the knife: A decivilizing process	Bristol	Literature review	N/A	N/A	**Marginalisation and Opportunity** Teenagers excluded from society Stigma increases chance of youth violence **Education** School exclusion – not receiving achievements 80% of young offenders have not completely secondary school
Nasr, IN et al 2010 Gender inequality in the risk of violence: material deprivation is linked to higher risk for adolescent girls.	South Wales (Cardiff, Newport, Swansea)	Cohort	699 residents 11–17 years 475 males and 224 females 12-month follow-up period	Rates and rate ratios	**Gender:** Injury rate for boys ranged from 10.9 to 22.3 per 1000 residents, and for girls 4.9 to 9.4. **Deprivation**: Associated with violence Evaluated rate ratio for injury resulting from violence for males and females, depending on area of deprivation e.g. RR for females in affluent area: 2.91 RR for females in deprived area: 6.31
Hayden, C 2010 Offending behaviour in care: is children’s residential care a ‘criminogenic’ environment?	England	Cohort	46 young people from 10 care homes 29 males and 17 females 12-month follow-up period	Percent of children offending	**Prevalence of offending** in 1-year: 76.1% **Types of offending**: violence against person most common (47.8%) Compared **school exclusion** and **number of placements** between offenders and non-offenders: 1 placement for non-offenders Numerous for offenders School exclusion prevalence similar: 40 and 44.8% for non-offenders and offenders, respectively **Females** are more likely to offend at an earlier age
Smith, D 2007 An investigation into causal links between victimization and offending in adolescents.	Edinburgh	Cohort	4300 secondary school students aged 12 years at start of study 5-year follow-up period	Correlation and ordinal regression models	**Analysed correlation coefficients for different variables** **Offending**: Type and how often Correlated with victimisation: 0.391 **Victimisation**: Asked about 5 types of victimisation (threatened/physical/weapon/steal directly or indirectly) and how often Correlated with offending: 0.421 **Bullying**: Prevalence and how often Correlated with victimisation: 0.372 and offending: 0.659 Victim of bullying correlated with victimisation 0.354 and offending 0.097 **Social class**: Information from parents’ surveys **Family structure**: Natural or adoptive parents Victimisation (0.246) and offending (0.276) higher for those in not with two birth parents **Neighbourhood**: Index of deprivation from 2001 census **Personality (impulsive behaviour**):5-point scale investigating risky behaviour Impulsivity linked more strongly to offending: 0.247, but linked to victimisation: 0.084 Risk taking linked to both: victimisation 0.294 and offending 0.545 **Relationship with parents**: 4-point verbal scale investigating conflict and supervision Conflicts with parents increase risk of victimisation and offending with correlation coefficients 0.257 and 0.266 **Risky spare-time activities**: Evaluated how often adolescents spent time with friends and what activities (e.g. cinema/clubs) Correlated with victimisation 0.171 and more strongly with offending 0.531
Densley, J et al 2011 Ganging up on gangs: Why the gang intervention industry needs an intervention.	London	Interview	51 self-ascribed gang members from London-based interventions 98% Black or Black British 88% males 12% females	N/A	**Stigma**: ‘you’re automatically stereotyped. It’s like all Black people are criminals’ **Police**: ‘No matter how many times police say they going to protect you, they’re not going to protect you’
Briggs, D 2010 ‘True stories from bare times on road’: Developing empowerment, identity and social capital among urban minority ethnic young people in London, UK.	London	Interview	34 gang members aged 12–24 years 19 females and 15 males Majority were Black African, 5 Black British, 5 mixed-race	N/A	Believed **school achievements** were unattainable **Pressure from peers** to adopt particular lifestyle **Stigma** from spending times in groups Attitudes not limited to **boys** Limited range of **employment** opportunities ‘Cycle of **retaliation’** Gang members expressed their lack of motivation to complete **education** Found no difference in **gender** roles
Densley, JA et al 2015 We’ll show you gang’: The subterranean structuration of gang life in London	London (6 boroughs with high serious violence rates)	Interview	69 self-ascribed gang members and associates Aged 13–34 years 77% male 93% Black or Black British	N/A	Reported high rates of violent **victimisation** **Marginalisation**: Described themselves at ‘urban outcasts’ ‘the odds are against us’ **Deprivation**: ‘No jobs, no opportunities’ High youth unemployment **Household criminality**: ‘if you’re family, you’re part and parcel of it’ **Youths** are more likely to be tempted by short-term gains Many gang members experience **victimisation**
Wood, JL et al 2017 Differentiating Gang Members, Gang Affiliates, and Violent Men on Their Psychiatric Morbidity and Traumatic Experiences.	UK	Case-control	1539 males Aged 19–30 years	Logistic regression analyses, and counts and percentages	Compared gang members, affiliates, and violent men assessing: **Psychosis** (questionnaire) – depression, suicide, self-harm (higher in gang members and affiliates compared to violent men) Alcohol and drug use Traumatic experiences (similar across all groups) Gang members are more likely to be placed into **care** by local authority
Barlas, J et al 2006 Weapons carrying in British teenagers: The role of personality, delinquency, sensational interests, and mating effort.	UK	Cross-sectional	121 Aged 11–18 59 females 62 males 95.9% White	Item factor analysis and binomial logistic regression	**Juvenile Attitude Towards Weapon Scale** Reasons and frequency of weapon carrying Self-report Early **Delinquency** Instrument Investigate variety of criminal offences **Sensational interest** Questionnaire **Mating Effort** Scale Looked at SES, living arrangements, age, and gender in relation to weapon carrying Prevalence of weapon carrying similar between different **genders** **Men** more likely to use weapon to injure As **age** increases likelihood of weapon possession increases
Alleyne, E et al 2016 Psychological and behavioural characteristics that distinguish street gang members in custody	UK	Case-control	188 individuals from youth offending institution Aged 16–18 years	ANOVA, means, standard deviations, and correlation coefficients	**Compared gang youth to non-gang youth** divided into four categories: 1) self-identified gang and meeting Eurogang definition 2) self-identified not meeting Eurogang definition 3) Matching Eurogang definition 4) non-gang youth Evaluated **reasons for gang involvement** Evaluated **group crime**: higher in gang youth **Moral Disengagement** **Hypermasculinity** Values Questionnaire Attitudes toward formal authority scale
Hansen, K. 2003 Education and the Crime-Age Profile	England and Wales	Case-control	2.529 males Aged 16–25 years	Percentage and Probit models	**Comparing those who left school at age 16 to those who remained in school** Looked at parent and family contact, household criminality, and school truancy
Alleyne, E et al 2014 Denying humanness to victims: How gang members justify violent behavior.	London	Case-control	189 participants from 4 youth centres and 1 secondary school Aged 12–25 152 males 37 females	ANOVA, means, standard deviation, and percentages	Looked at demographics of individuals Assessed gang membership: 1) gang members 2) affiliates 3) non-gang youth Assessed **violent crime** and mechanisms of **moral disengagement** Gang members more likely to show **psychological** issues and be involved in violent crime No difference found between **gender** roles. **Young people** at high-risk of violence **Age**: No significant difference between non-gang (mean age = 15.08) and gang members (M = 15.29)
Alleyne, E et al 2010 Gang involvement: psychological and behavioral characteristics of gang members, peripheral youth, and nongang youth.	London	Cross-sectional	797 school students Aged 12–18 556 males 231 females	ANOVA, reliability analysis, and MANCOVA	Individuals divided into gang youth, peripheral youth, and non-gang youth Assessing gang membership, age, gender, ethnicity **Delinquency**: Higher in gang members compared to non-gang youth Perception of out of group threat: **Social status** scale: Status more important to gang members Mechanisms of **moral disengagement**: Gang members displace responsibility, attribute blame and use euphemistic language Attitude toward formal **authority** scale: Gang youth scored higher on **anti-authority attitudes** No difference found between **gender** roles **Age** varies with gang membership
Bailey, S et al 2001 The social background and nature of “children” who perpetrate violent crimes: A UK perspective.	UK	Cross-sectional	20 male adolescents convicted of homicide Aged 10–17	Counts and percentages	35% had **prior history of offending** 25% had been sexually or physically **abused** All had experienced neglect or **parental separation** All had history of disruptive behaviour in **school**
Falshaw et al 1997 Adverse childhood experiences and violent acts of young people in secure accommodation	Birmingham	Cross-sectional	79 participants from a care home 42% females 58% males	Counts and percentages	91% had history of pl**acement in care** (range 1–30 placements) Assessed violent crimes (51% carried weapons and 77% were involved in violent crime) Average age of offending: **11 years 7 months** 70/79 had **behaviour problems** **Males** commit offence earlier than girls

## Appendix 3

Table of SWiM checklist used as guidance to report methods of the narrative synthesis conducted. Includes where information can be found in manuscript by page number

SWiM covers reporting of the key features of synthesis including how studies are grouped, synthesis methods used, presentation of data and summary text, and limitations of the synthesis.

**Table:**

SWiM reporting item	Item description	Page in manuscript
Methods		
1 Grouping studies for synthesis	1a) Provide a description of, and rationale for, the groups used in the synthesis (eg, groupings of populations, interventions, outcomes, study design)	7
	1b) Detail and provide rationale for any changes made subsequent to the protocol in the groups used in the synthesis	N/A
2 Describe the standardised metric and transformation methods used	Describe the standardised metric for each outcome. Explain why the metric(s) was chosen and describe any methods used to transform the intervention effects, as reported in the study, to the standardised metric, citing any methodological guidance consulted	N/A
3 Describe the synthesis methods	Describe and justify the methods used to synthesise the effects for each outcome when it was not possible to undertake a meta-analysis of effect estimates	9 & 10
4 Criteria used to prioritise results for summary and synthesis	Where applicable, provide the criteria used, with supporting justification, to select the particular studies, or a particular study, for the main synthesis or to draw conclusions from the synthesis (eg, based on study design, risk of bias assessments, directness in relation to the review question)	10
5 Investigation of heterogeneity in reported effects	State the method(s) used to examine heterogeneity in reported effects when it was not possible to undertake a meta-analysis of effect estimates and its extensions to investigate heterogeneity	10
6 Certainty of evidence	Describe the methods used to assess the certainty of the synthesis findings	10–12
7 Data presentation methods	Describe the graphical and tabular methods used to present the effects (eg, tables, forest plots, harvest plots)	10 & 29–34
	Specify key study characteristics (eg, study design, risk of bias) used to order the studies, in the text and any tables or graphs, clearly referencing the studies included	
Results		
8 Reporting results	For each comparison and outcome, provide a description of the synthesised findings and the certainty of the findings. Describe the result in language that is consistent with the question the synthesis addresses, and indicate which studies contribute to the synthesis	17–22
Discussion		
9 Limitations of the synthesis	Report the limitations of the synthesis methods used and/or the groupings used in the synthesis and how these affect the conclusions that can be drawn in relation to the original review question	23 & 24

## Abbreviations

ACE: Adverse Childhood Experience
SES: Socioeconomic status
UK: United Kingdom
WHO: World Health Organization
WOE: Weight of Evidence
